# Fungal biotransformation of ezetimibe

**DOI:** 10.1080/13102818.2014.966948

**Published:** 2014-11-17

**Authors:** Irfan Pervaiz, Saeed Ahmad, Farhan Hameed Khaliq, Adeel Arshad, Muhammad Imran, Barkat Ali Khan, Aftab Ullah, Usman Ali, Kashif Iqbal, Muhammad Usman, Hafsa Bibi, Najm Ul Hassan Khan, Wajahat Mahmood

**Affiliations:** ^a^ Department of Pharmacy, COMSATS Institute of Information Technology, AbbottabadPakistan; ^b^ Faculty of Pharmacy and Alternative Medicine, Islamia University of Bahawalpur, BahawalpurPakistan; ^c^ Department of Pharmacy, Gomal University, Dera Ismail Khan, Pakistan; ^d^ Faculty of Pharmacy, University of Balochistan, Quetta, Pakistan; ^e^ Department of Pharmacy, Abasyn University, Peshawar, Pakistan; ^f^ Department of Chemistry, Gomal University, Dera Ismail Khan, Pakistan; ^g^ Islam College of Pharmacy, Sialkot, Pakistan

**Keywords:** biotransformation, ezetimibe, UDP-Glucuronyl transferase system, antihypercholesterolemic, *Beauvaria bassiana*, *Cunninghamella blakesleeana*

## Abstract

Structural transformation of ezetimibe was performed by fungi *Beauvaria bassiana* and *Cunninghamella blakesleeana*. The metabolites were identified by different spectroscopic techniques as (3R,4S)-1-(4-fluorophenyl)-3-((E)-3-(4-fluorophenyl) allyl)-4-(4-hydroxyphenyl) azetidin-2-one (2), (3R, 4S)-1-(4-fluorophenyl)-3-(3-(4fluorophenyl)-3-oxopropyl)-4-(4-hydroxyphenyl) azetidin-2-one (3), (3R,4S) 1-(4-fluorophenyl)-3-(3-(4-fluorophenyl) propyl)-4-(4-hydroxyphenyl) azetidin-2-one (4) and (2R,5S)-N, 5-bis (4-fluorophenyl)-5-hydroxy-2-(4-hydroxybenzyl) pentanamide (5). This study displays two important features of these fungi, viz., their ability to metabolize halogenated compounds, and their capacity to metabolize drugs that are targets of the UDP-Glucuronyl Transferase System, a phenomenon not commonly observed.

## Introduction

Ezetimibe is a first prototype drug that represents the latest class of antihypercholesterolemic and lipid modifying agents exerting its effects via blocking cholesterol absorption.[1] It is believed to inhibit lipid absorption by preventing dietary and billiary cholesterol to move across the intestinal wall without influencing the absorption of fat soluble vitamins, triglycerides and bile acids.[2–4]

Ezetimibe localizes in the brush border of the small intestinal enterocytes and decreases the uptake of cholesterol into the enterocytes. This has the net effect of inhibiting cholesterol absorption from the intestinal lumen facilitating its excretion.[5]

Pre-clinical studies established that ezetimibe was glucuronidated to a single metabolite localized at the intestinal wall, where it prevented cholesterol absorption.[6] It was found that enterohepatic recirculation of ezetimibe and/or its glucuronide ascertained repeated delivery to the site of action and restrained peripheral exposure.[7] Ezetimibe has no effect on the action of major drug metabolizing enzymes (CYP450), which reduces the possible drug–drug interactions.[5]

Ezetimibe and its active metabolite are highly bound to human plasma proteins (90%). Ezetimibe is primarily metabolized in the liver and in the small intestine via glucuronide conjugation with subsequent renal and biliary excretion.[6] Both the parent compound and its active metabolite are eliminated from plasma with a half-life of approximately 22 h allowing for once daily dosing. Ezetimibe is devoid of substantial inhibitory or inductive effects on cytochrome P-450 isoenzymes which explains its limited number of drug interactions.[7,8]

The main objective of this study was to find a microbial metabolic system that mimics mammalian metabolism closely regarding halogenated drugs to aid the pre-clinical studies and exploring the enzymatic potential of microbes for drugs excreted via glucuronidation, thus, revealing the presence of a UDP-Glucuronyl transferase system, as well as its proposed activity in microbial biotransformation of pharmacologically active compounds. In addition, discovery of novel metabolites that are pharmacologically superior and less toxic in comparison to their predecessors [9] was also identified as an aim of the study.

## Materials and methods

### Instrumentation

Electron impact mass spectrometry (EI-MS) was performed on Jeol JMS-600 H mass spectrometer. The ^1^H-NMR, ^13^C-NMR, ^1^H,^1^H-COSY 45°, HMBC, HSQC, NOESY spectra were recorded in deuterated CDCl3 on Bruker Avance-500, 400, 300 and 100 MHz instruments. Chemical shift (δ) in ppm and coupling constants (*J*) are in Hz, related to Tetramethylsilane (TMS) which was used as an internal standard. Ultraviolet (UV) experiments were recorded in spectroscopic grade methanol on a Shimadzu UV 240 (Shimadzu CorporationTokyo, Japan). Infrared (IR) spectra were measured in chloroform, as KBr disc on Shimadzu FT IR-8900 spectrophotometer or JASCO A-302 (JASCO International Co. Ltd., Japan).

### Microbial cultures

The microbial cultures were purchased from American Type Culture Collection (ATCC), USA; Northern Regional Research Laboratories (NRRL) USA and Institute of Fermentation, Osaka, Japan (IFO). These microbial cultures were maintained on Sabouraud Dextrose Agar media (SDA) slants and incubated at 4 °C before use.

### General protocol for media preparation

Two-day-old spores of each fungus were aseptically transferred into broth medium flasks containing 100 mL of freshly prepared autoclaved medium. The seed flasks thus obtained were incubated on a shaker at 30 °C for two days. These broth cultures were inoculated aseptically into 60 media flasks of 250 mL each containing 100 mL of medium, and fermentation was continued for further 24 h. In brief, 2000 mg ezetimibe designated as compound 1 was diluted with 60 mL methanol, the resulting solution was evenly distributed among 60 conical flasks having shake cultures and the fermentation was continued for 12 days for *Beauvaria bassiana* and nine days for *Cunninghamella blakesleeana*. Fifteen litres of ethyl acetate was used to wash, filter and extract the mycelia. The extracts were dried over anhydrous sodium sulphate and concentrated *in vacuo* to afford gums that were adsorbed on equal quantities of silica gel and eluted with various solvent gradients of petroleum ether, ethyl acetate and methanol.

## Results and discussion

Ezetimibe (compound 1) (see Table 1) was fermented in broth cultures of *B. bassiana* and *C. blakesleeana* for 12 and nine days, respectively. Ethyl acetate was used to extract the parent compound and the metabolites from mycelia and the components were separated using silica gel columns. The fermentation resulted in the production of compounds 2 and 3 (see Tables 2 and 3) in 15.8% and 6.5% yields from *B. bassiana*, and compounds 4 and 5 (see Tables 4 and 5) in 5.2% yield from *C. blakesleeana*, respectively. Compound 2 was obtained from sub-column via further elution through addition of 42% ethyl acetate/petroleum ether. The remaining compound 1 was recovered unchanged. Compounds 4 & 5 were eluted and purified via similar chromatographic techniques as mentioned above.

**Table 1.** Compound (1). ^1^H-NMR (100 MHz, CDCl_3_) and ^13^C-NMR (600 MHz, CDCl_3_) chemical shift assignments of ezetimibe (1) (3*R*, 4S)-1-(4-fluorophenyl)-3-[(3S)-3-(4-fluorophenyl)-3-hydroxypropyl]-4-(4-hydroxyphenyl) azetidin-2-one (1).

Position	Integration	^1^H (Chemical shift in ppm)	Multiplicity (J Hz)	^13^C (Chemical shift in ppm) HSQC	Nature
1	–	–	–	–	–
2	–	–	–	165.8	–
3	1H	3.05	m	58.8	CH
4	1H	4.84	d (2.4)	63.0	CH
5	–	–	–	142.8	–
6,6′	2H	7.15	m	120.7	CH
7,7′	2H	7.15	m	115.4	CH
8	–	–	–	161.7	–
9	2H	1.64	m	24.6	CH_2_
10	2H	1.85	m	36.1	CH_2_
11	1H	4.46	m	72.6	CH
12	–	–	–	132.4	–
13,13′	2H	7.32	m	128.7	CH
14,14′	2H	7.15	m	115.3	CH
15	–	–	–	162.0	–
16 (OH)	1H	5.45	d (4.5)	–	–
17	–	–	–	129.2	–
18,18′	2H	7.15	d (8.6)	127.9	CH
19,19′	2H	6.72	d (8.6)	115.9	CH
20	–	–	–	156.8	–
21 (OH)	1H	9.45	br s	–	–

**Table 2.** Compound (2). ^1^H-NMR (100 MHz, CDCl_3_) and ^13^C-NMR (600 MHz, CDCl_3_) chemical shift assignments of (3R,4S)-1-(4-fluorophenyl)-3-((E)-3-(4-fluorophenyl) allyl)-4-(4-hydroxyphenyl) azetidin-2-one (2).

Position	Integration	^1^H (Chemical shift in ppm)	Multiplicity (J Hz)	^13^C (Chemical shift in ppm) HSQC	Nature
1	–	–	–	–	–
2	–	–	–	165.8	C
3	1H	3.05	m	58.8	CH
4	1H	4.84	d (2.4)	63.0	CH
5	–	–	–	142.8	C
6,6′	2H	7.15	m	120.7	CH
7,7′	2H	7.15	m	115.4	CH
8	–	–	–	161.7	C
9	2H	2.68, 2.90	m	34.6	CH_2_
10	1H	6.25	m	116.1	CH
11	1H	6.80	d (14.6)	122.6	CH
12	–	–	–	128.6	C
13,13′	2H	7.70	m	131.4	CH
14,14′	2H	7.34	m	115.2	CH
15	–	–	–	164.1	C
16	–	–	–	127.2	C
17,17′	2H	7.15	d (8.6)	127.9	CH
18,18′	2H	6.78	d (8.6)	116.0	CH
19	–	–	–	157.2	C
20 (OH)	1H	9.45	br s	–	–

**Table 3.** Compound (3). H-NMR (100 MHz, CDCl_3_) and ^13^C-NMR (600 MHz, CDCl_3_) chemical shift assignments of (3R,4S)-1-(4-fluorophenyl)-3-(3-(4-fluorophenyl)-3-oxopropyl)-4-(4-hydroxyphenyl) azetidin-2-one (3).

Position	Integration	^1^H (Chemical shift in ppm)	Multiplicity (J Hz)	^13^C (Chemical shift in ppm) HSQC	Nature
1	–	–	–	–	–
2	–	–	–	166.8	–
3	1H	3.06	m	59.0	CH
4	1H	4.8	d (2.3)	63.3	CH
5	–	–	–	142.8	–
6,6′	2H	7.09	m	120.7	CH
7,7′	2H	7.09	m	115.4	CH
8	–	–	–	161.7	–
9	2H	1.67	dd (2.11,2.19)	24.9	CH_2_
10	2H	1.87	t (2.03)	36.4	CH_2_
11	–	–	–	195.5	C
12	–	–	–	125.4	–
13,13′	2H	8.12	m	131.2	CH
14,14′	2H	7.30	m	115.1	CH
15	–	–	–	161.5	–
16 (O)	–	–	–	–	–
17	–	–	–	129.2	–
18,18′	2H	7.15	d (8.5)	127.9	CH
19,19′	2H	6.72	d (8.5)	115.9	CH
20	–	–	–	156.8	–
21 (OH)	1H	9.45	br s	–	–

**Table 4.** Compound (4). ^1^H-NMR (100 MHz, CDCl_3_) and ^13^C-NMR (600 MHz, CDCl_3_) chemical shift assignments of (3R,4S) 1-(4-fluorophenyl)-3-(3-(4-fluorophenyl) propyl)-4-(4-hydroxyphenyl) azetidin-2-one (4).

Position	Integration	^1^H (Chemical shift in ppm)	Multiplicity (J Hz)	^13^C (Chemical shift in ppm) HSQC	Nature
1	–	–	–	–	–
2	–	–	–	165.8	C
3	1H	3.05	m	58.8	CH
4	1H	4.84	d (2.4)	63.0	CH
5	–	–	–	142.8	C
6,6′	2H	7.15	m	120.7	CH
7,7′	2H	7.15	m	115.4	CH
8	–	–	–	161.7	C
9	2H	2.10	m	34.6	CH_2_
10	2H	1.68	m	116.1	CH_2_
11	2H	2.21	m	122.6	CH_2_
12	–	–	–	130.5	C
13,13′	2H	7.32	m	135.7	CH
14,14′	2H	7.15	m	115.3	CH
15	–	–	–	162.0	C
16	–	–	–	127.2	C
17,17′	2H	7.15	m	127.9	CH
18,18′	2H	6.72	m	116.0	CH
19	–	–	–	157.2	C
20 (OH)	1H	9.45	br s	–	–

**Table 5.** Compound (5). ^1^H-NMR (100 MHz, CDCl_3_) and ^13^C-NMR (600 MHz, CDCl_3_) chemical shift assignments of (2R,5S)-N, 5-bis (4-fluorophenyl)-5-hydroxy-2-(4-hydroxybenzyl) pentanamide (5).

Position	Integration	^1^H (Chemical shift in ppm)	Multiplicity (J Hz)	^13^C (Chemical shift in ppm) HSQC	Nature
1 (NH)	1H	9.78	br s	–	–
2	–	–	–	174.4	–
3	1H	2.51	m	58.8	CH
4	2H	2.51, 2.33	m	63.0	CH
5	–	–	–	136.9	–
6,6′	2H	7.57	m	122.5	CH
7,7′	2H	7.10	m	114.8	CH
8	–	–	–	161.7	–
9	2H	1.51	m	28.6	CH_2_
10	2H	1.68	m	36.8	CH_2_
11	1H	4.46	m	73.0	CH
12	–	–	–	132.4	–
13,13′	2H	7.32	m	128.8	CH
14,14′	2H	7.12	m	115.3	CH
15	–	–	–	162.0	–
16 (OH)	1H	5.38	d (4.5)	–	–
17	–	–	–	133.2	–
18,18′	2H	6.98	d (8.6)	133.0	CH
19,19′	2H	6.60	d (8.6)	114.9	CH
20	–	–	–	155.8	–
21 (OH)	1H	9.05	br s	–	–

**Figure 1.**
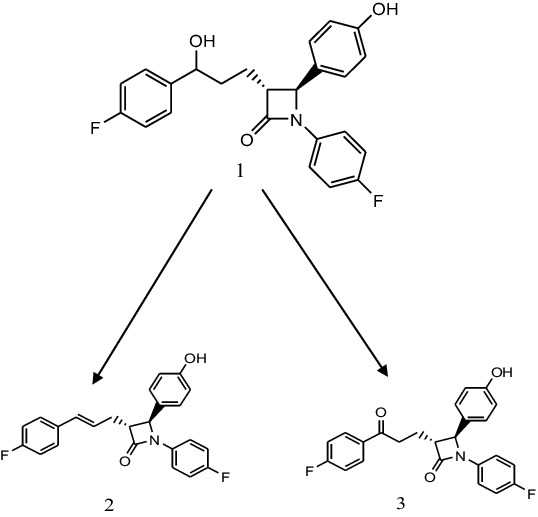
Biotransformation of ezetimibe (1) by *Beauvaria bassiana* into compound (2) and compound (3).

**Figure 2.**
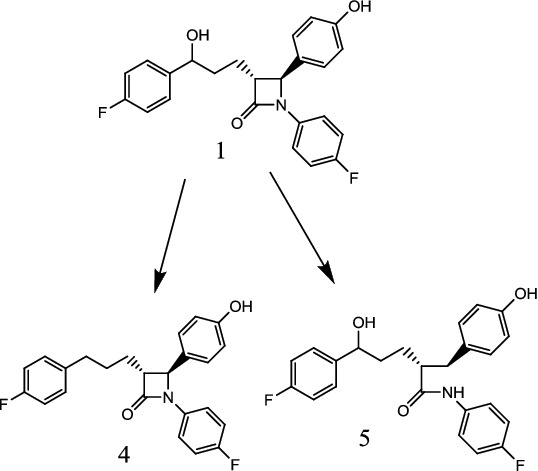
Biotransformation of ezetimibe (1) by *Cunninghamella blakesleeana* into compound (4) and compound (5).

The chemical structures (Figures 1 and 2) were elucidated on the basis of their spectra of MS, ^1^H and ^13^C NMR (Supplementary data) and compared with the data previously reported.[10–14]

### Biotransformation of Ezitimibe (1) by B. bassiana

Incubation of ezetimibe (1) with *B. bassiana* yielded two known metabolites, namely 4-((2S,3S)-1-(4-fluorophenyl)-3-((S)-3-(4-fluorophenyl)-3-hydroxypropyl)azetidin-2-yl)phenol (2) and 1-(4-fluorophenyl)-3-(3-(4-fluorophenyl)propyl)-4-(4-hydroxyphenyl)azetidin-2-one (3). The proposed biotransformation pathway is presented in Figure 1. These compounds have previously been obtained via synthetic modifications of azetidinone nucleus.[2,3,7–10]

#### Compound (2)

[(3R,4S)-1-(4-fluorophenyl)-3-((E)-3-(4-fluorophenyl) allyl)-4-(4-hydroxyphenyl) azetidin-2-one] had the molecular formula of C_24_H_19_F_2_NO_2_ and it possessed the molecular weight of 391.4100 which was inferred from EI-MS studies as the metabolite displayed molecular ion peak at *m*/*z* 391.1385 (calcd 391.1417), which was 18.0 amu less than the parent compound, thus, suggesting the possible removal of a–OH group. Metabolite 2 showed strong absorption at 242 nm in UV spectrum thus indicating the presence of α, β unsaturated ketonic function. The IR spectrum of the metabolite showed loss of one absorption signal at 3310 cm^−1^. The ^13^C– and ^1^H-NMR spectra revealed the absence of the hydroxyl group at the 16th position. The ^13^C-NMR spectra (BB, DEPT-90 and DEPT-135) exhibited the resonances for 1 methylene, 16 methine and 7 quarternary carbon atoms. EI-MS *m*/*z*: 391.1385 (calcd 391.1417) IR (CHCl_3_) ν_max_ cm^−1^: 3483 (OH), 1661 (C=C), 1712 (C=O).

#### Compound (3)

[(3R,4S)-1-(4-fluorophenyl)-3-(3-(4-fluorophenyl)-3-oxopropyl)-4-(4-hydroxyphenyl) azetidin-2-one] had the molecular formula of C_24_H_19_F_2_NO_3_. EI-MS experiments indicated an M^+^ peak at 407.1250 (calcd 407.1181). The molecular weight was estimated to be 407.4081 g/mol. This compound is UV active. The UV analysis showed absorbance at 236 nm for a ketonic group. The IR spectrum of the compound showed sharp absorption for a ketone at 1707 cm^−1^ with the disappearance of hydroxy absorption peak (3310 cm^−1^). The ^1^H-NMR spectrum pointed out the disappearance of the –OH signals. The ^13^C-NMR spectra (BB, DEPT-90 and DEPT-135) displayed the resonances for 4 methylene, 13 methine and 7 quarternary carbon atoms. The mass spectrum showed that the mass of the compound was 407.4081 g/mol which were 1 amu less than the substrate showing loss of one hydrogen atom. Henceforth, the metabolite was characterized as 1-(4-fluorophenyl)-3-(3-(4-fluorophenyl) propyl)-4-(4-hydroxyphenyl) azetidin-2-one (3) EI-MS *m*/*z*: 407.1250 (calcd 407.1181) IR (CHCl_3_) ν_max_ cm^−1^: 3447 (OH), 1718 (C=O).

### Biotransformation of Ezeitimibe (1) by C. blakesleeana

Biotransformation of ezetimibe (1) with *C. blakesleeana* yielded two known metabolites (3R,4S) 1-(4-fluorophenyl)-3-(3-(4-fluorophenyl) propyl)-4-(4-hydroxyphenyl) azetidin-2-one (4) and (2R, 5S)-N, 5-bis (4-fluorophenyl)-5-hydroxy-2-(4-hydroxybenzyl) pentanamide (5). The proposed biotransformation pathway is presented in Figure 2. These compounds have previously been reported via synthetic modifications of azetidinone nucleus.[7,8,10–16]

#### Compound (4)

[(3R,4S) 1-(4-fluorophenyl)-3-(3-(4-fluorophenyl) propyl)-4-(4-hydroxyphenyl) azetidin-2-one] had the molecular formula of C_24_H_21_F_2_NO_2_ and it possessed the molecular weight of 393.4258, which was deduced from EI-MS studies, as the metabolite displayed molecular ion peak at *m*/*z* 393.1540 (calcd 393.1574), which was 18.0 amu less than the parent compound, thus, suggesting the possible removal of a–OH group. The IR spectrum of the metabolite showed a loss of one absorption signal at 3310 cm^−1^. The ^13^C– and ^1^H-NMR spectra revealed the absence of the –OH group at the 11th position and the appearance of additional methylene signal. The ^13^C-NMR spectra exhibited the resonances for 3 methylene, 14 methine and 7 quarternary carbon atoms. EI-MS *m*/*z*: 393.1540 (calcd 393.1574) IR (CHCl_3_) ν_max_ cm^−1^: 3481 (OH), 1710 (C=O).

#### Compound (5)

[(2R,5S)-N, 5-bis (4-fluorophenyl)-5-hydroxy-2-(4-hydroxybenzyl) pentanamide] had the molecular formula of C_24_H_23_F_2_NO_3_. EI-MS experiments indicated an M^+^ peak at 411.1646 (calcd 412.1680). The molecular weight was estimated to be 411.4411. This compound is UV active. The mass spectrum showed that the mass of the compound of 411.4411 was approximately 1 amu higher than the substrate showing addition of one hydrogen atom. Henceforth, the metabolite was characterized as 1-(4-fluorophenyl)-3-(3-(4-fluorophenyl) propyl)-4-(4-hydroxyphenyl) azetidin-2-one (3). The ^1^H NMR spectral data of metabolite 5 disclosed additional signals at 2.81 and 9.75 ppm. The signal at 9.75 ppm, which is not observed with ezetimibe, conforms to an exchangeable proton and is spatially close to protons at 6 and 6′ positions, consequently it is assigned as –NH group. This could mean that the metabolite 5 has an extra –CH_2_ group at 38.1 ppm as compared to that of ezetimibe, 1H–1H correlation revealed that the C4 is now –CH_2_ and linked to two protons at 2.81 and 2.94 ppm and no more CH as it is in ezetimibe. The ^13^C-NMR spectra (BB, DEPT-90 and DEPT-135) confirmed the presence of 14 methine, three methylene and seven quaternary carbon atoms. EI-MS m/z: 411.1646 (calcd 412.1680).IR (CHCl_3_) ν_max_ cm^−1^: 3466 (OH), 1707 (C=O).

## Conclusion

Microbial biotransformation of ezetimibe resulted in four known compounds illustrating the enzymatic capacities of *B. bassiana* and *C. blakesleeana*. It also shows the ability of these strains to metabolize synthetic compounds in addition to their ability to metabolize natural compounds.[11] These fungi also display the ability to biotransform halogenated compounds, a difficult process as halogenated compounds often exhibit antimicrobial/antifungal properties.[12] These experiments shed light upon an important aspect that the fungi utilized have metabolising capacity for drugs metabolized via the UDP-Glucuronyl transferase system, as those drugs that are targets of CYP450 are more commonly known targets of fungal metabolism.[13]

Thus, our experiments open the door for possibility of designing new analogues from synthetic drugs via biotransformation. These derivatives might show enhanced activity in terms of better pharmacokinetic profile. Alternatively these metabolites may exhibit off-target pharmacology.
